# Exploring determinants of hand hygiene among nursing students: A theory of planned behavior approach

**DOI:** 10.1186/s12912-024-02062-0

**Published:** 2024-06-17

**Authors:** Gül Bülbül Maraş, Elem Kocaçal

**Affiliations:** 1 Elderly Care Program, Vocational School of Health Services, İzmir Demokrasi University, İzmir, Turkey; 2 Fundamentals of Nursing, Faculty of Health Sciences, İzmir Demokrasi University, İzmir, Turkey

**Keywords:** Theory of Planned Behavior, Hand Hygiene Scale, Hand Hygiene Behavior, Nursing Students

## Abstract

**Background:**

This study aims to assess the hand hygiene behavior of nursing students and identify the factors influencing this behavior through the “Scale for Assessment Hand Washing Behavior in the Frame of Theory of Planned Behaviour (SAHBTPB)”.

**Methods:**

This descriptive and cross-sectional study was undertaken at the nursing departments of the university’s faculty of health sciences in İzmir, Turkey between 2021 and 2022. A total of 240 nursing students were recruited as participants for this study. Data were collected with the SAHBTPB. The data was analyzed using descriptive statistics, the Chi-square test, and correlation analysis in the SPSS 21.0 program (*p* < .05).

**Results:**

Participation rate was 74.76%. The mean age of the students was 20.59 ± 1.59 years and 69.9% were woman. The nursing students’ total mean score of SAHBTPB was 147.5 ± 14.0 (min = 94; max = 176). There was a positively significant association between the total score and students’ gender, graduate level, and hand hygiene education status. There was no significant difference in scale total score mean based on the existence of dermatological problems on the students’ hands or their frequency of hand hygiene (*p* > .05).

**Conclusion:**

The mean scores of nursing students on the SAHBTPB were found to be at a good level. The sub-dimension “intention” was identified as an effective factor in predicting the hand hygiene behavior of the students. The findings have the potential to positively impact nursing education by increasing awareness among students and offering valuable insights for nurses and educators.

## Background

Healthcare-associated infections (HAIs) reduce the quality of care, puts patient safety at risk, and increase costs. Almost 30–50% of HAIs can be prevented with hand hygiene [1–3]. Therefore, strategies for keeping hands clean are extremely crucial for safe healthcare delivery in healthcare institutions [1–4]. The World Health Organization (WHO) guidelines clearly emphasize the significance of hand hygiene and provide instructions on proper handwashing with soap and water or using hand antiseptic [3]. Although hand hygiene may appear a simple and easy procedure, hand hygiene compliance rates among healthcare professionals in healthcare institutions are still far from the desired levels [4–7]. Hence, a series of initiatives and global campaigns are being conducted to improve adherence to hand hygiene practices [8].

Behavioral theories have been widely employed to investigate various health-related behaviors. Among the various theories employed, The Theory of Planned Behavior (TPB) is one of the most common theories used to investigate individual behaviors. It has been also utilized to anticipate hand hygiene behaviors in high-risk populations like food handlers and healthcare workers [6, 9, 10]. The TPB was proposed by Icek Ajzen (1991) and explains the motivation behind intentional behaviors [11–13]. The theory assumes that the primary determinant of intentional behaviors, such as hand hygiene, is the intention to perform the behavior. Intention is considered the precursor of behavior, and the stronger the intention, the more likely the behavior is assumed to be [9]. Intention is determined by attitude, subjective norm, and perceived behavioral control [10, 14]. Attitude represents beliefs about the consequences of a behavior and their positive or negative evaluation. Subjective norm indicates the influence of significant others outside the institution on an individual’s hand hygiene behavior. It also represents the perceived social pressure towards the behavior. Perceived behavioral control reflects beliefs about access to resources and opportunities required to perform a behavior [15]. The TPB suggests that the intention is influenced by indirect predictors named behavioral, normative, and control beliefs. Beliefs about the outcomes of behavior are based on perceiving the advantages and disadvantages of performing a specific behavior [6]. Normative beliefs express the personal probability of whether an individual wants or does not want a given behavior in relation to a specific normative referent. In other words, it refers to the perception of the expectations of normative individuals within the institution regarding the individual’s performance of behavior. Control beliefs are related to various factors (time, cost, available infrastructure, etc.) that facilitate or hinder a behavior [14, 16]. In general, intention of an individual to engage in a specific behavior is expected to be stronger when they have more positive attitudes and subjective norms, and a higher level of perceived control over the behavior. According to the theory, attitudes determine intention, and intention, in turn, is a determinant of behavior [10, 12, 17].

Nursing students are regarded as future healthcare professionals during their education. Their direct contact with patients in clinical practice settings during treatment and care may increase the risk of cross-contamination [18, 19]. Integrating theory and practice is a crucial way to learn effective hand hygiene practices during nursing education [2]. Even when students have demonstrated sufficient theoretical and practical knowledge, they may appear unwilling or unable to apply what they know in clinical settings [20]. Hand hygiene compliance is a complex behavior and is affected by many factors such as workload, lack of role models, lack of equipment or information, overconfidence in one’s own abilities [4, 21]. Studies on nursing students’ hand hygiene practices have shown that compliance is influenced by individual characteristics like attitude, perception, belief, and knowledge, along with external factors such as the hand hygiene behaviors of healthcare professionals in clinical settings [22–26]. Understanding the factors influencing nursing students’ hand hygiene practices will guide future research, clinical applications, and the development of education strategies. There are many studies in the literature that have assessed nurses’ hand hygiene behavior using the TPB [6, 13, 23]. But there is a limited number of studies examining the hand hygiene behavior of nursing students in terms of the TPB [24, 27, 28].

## Methods

### Aim

The objective of this study was to examine the hand hygiene behavior of nursing students at a university’s faculty of health sciences and affecting factors using the SAHBTPB. Our study is the first study in Turkey where the TPB was used to determine hand hygiene behavior and affecting factors.

### Setting and sample

This descriptive and cross-sectional study took place in the nursing department, faculty of health sciences at a state university in Western Turkey. The study was conducted during the spring semester of the 2021–2022 academic year.

The population consisted of a total of 321 nursing students studying in the first, second, third, and fourth grades. No sampling method was employed since the study aimed to encompass the entire population. 36 nursing students were absent or received medical reports, 24 students refused to participate in the study, and 21 students were excluded due to incomplete data collection forms. Volunteer students with no communication problems formed the study sample. The exact sample size was determined by using the G-Power 3.1.9.2 software package. According to the power analysis results, the required sample size for the study, with an effect size of 0.40 and epsilon of 1 for the analysis of variance in repeated measures, to achieve a 95% test power at a 95% confidence level, was determined to be a minimum of 230 individuals. In total, the study included 240 students, resulting in a participation rate of 74.76%.

### Data collection tools

Data were collected using the “Students Identification Form” and the SAHBTPB. The form developed by researchers in line with the comprehensive literature consists of 15 questions such as age, gender, graduation level, hand hygiene training status, frequency of performing hand hygiene, and the presence of a dermatological problem in the hand [9, 15, 17, 24, 29–31].

SAHBTPB was developed by Maraş (2007) [17–19]. The scale was developed to determine the motivational factors (attitude, belief) and knowledge of healthcare professionals affecting hand hygiene behavior. The scale was suggested to use in healthcare professionals and students. The scale consists of 46 items and eight sub-dimensions: Beliefs about the consequences of hand hygiene, subjective norm, normative beliefs, control beliefs, perceived control, attitude, intention and knowledge. The first seven sub-dimensions of the scale are Likert type and have four categories. The four-point Likert scale is “Strongly agree” 4 points, “agree” 3 points, “disagree” 2 points, “Strongly disagree” 1 point. The “knowledge” sub-dimension is evaluated as yes (3), I don’t know (2), and no (1). The 8th, 9th, 10th, 11th, 12th, 31st, 33rd, 34th items in the scale are negative items. The scores of these items were reverse coded. The lowest score achievable from the scale is 46 and the highest score is 176. A high score indicates a positive and strong motivation for hand hygiene behavior [17]. In Maraş’s (2007) study, the overall Cronbach’s alpha coefficient of the scale was found to be 0.79 and the total item correlation coefficient was 0.88 [17]. In this study, the overall Cronbach’s alpha coefficient of the scale was found to be 0.93.

### Data collection

After a verbal explanation of the study goals, informed consent form was obtained from the students who agreed to participate in the study. Students filled out the data collection forms under the supervision of the researchers. Data collection was carried out in the classroom at the same time to prevent interaction among the students. The completion of the forms took approximately 10 min.

### Data analysis

Statistical Package for Social Science (SPSS) 21.0 package program was used for the data analysis. The adequacy of the sample size was calculated using Power Analysis. Descriptive statistics are shown with mean, standard deviation, minimum and maximum values, and data for categorical variables are shown with frequency and percentage. The normality of the data distribution was evaluated with the Shapiro-Wilk test. Non-parametric analyzes such as Chi-square test, Correlation analysis, Mann-Whitney U test and Kruskal-Wallis test were used in the statistical analyses. *p* < .05 was accepted as the significance level.

### Ethical considerations

While ethical approval was received from the Non-Interventional Clinical Research Ethics Committee of the Health Science University to conduct the study (Approval date and no. 2021/3–11). The study was conducted in accordance with the principles of the Declaration of Helsinki. The participants were informed of the study’s goal and given the assurance that their nonparticipation or withdrawal would not affect them negatively. Written consent was obtained from all participants.

## Results

### Characteristics of students

The mean age of the students was 20.59 ± 1.59 years (min: 18, max: 30), and 69.9% were woman. All the students were single, and 94.1% of them have graduated from high school. Of the students, 36.7% were first-grade, 26.7% were second-grade, 17.4% were third grade, and 19.2% were fourth-grade students (Table 1).

**Table 1:** Characteristics of the nursing students (*n* = 240)

Features	X̅ ± SD	
**Age**		
Mean Minimum Maximum	20.59 ± 1.59 18 30	
	Number	%
**Gender**		
Woman Male	167 73	69.9 30.1
**Marital status**		
Single	240	100
**Graduated school**		
Health college High school Associate degree	5 226 9	2.1 94.1 3.8
**Level of grade**		
First grade Second grade Third grade Fourth grade	88 64 42 46	36.7 26.7 17.4 19.2

X̅ ±SD = Total score mean ± Standard deviation

### Nursing students’ opinions about hand hygiene

Hand hygiene was identified by 42.9% of nursing students as the most effective method for preventing HAIs, followed by sterilization (28.7%), and asepsis/antisepsis (16.7%). It was determined that 82.4% of the students reported that disposable paper towels were the most effective method for drying hands. Although 77.1% of the students received training on hand hygiene, 24.2% stated that they felt the need for further training on hand hygiene. Among students, 59.6% using general liquid soap and water in their daily lives and 36.2% reported performing hand hygiene 9–12 times, 23.8% 13–17 times daily. After hand hygiene, 72.9% of students confirmed they always dried their hands. 13.7% of students reported having a dermatological problem on their hands. As barriers to hand hygiene, 36.6% of the students stated that there was not enough hand hygiene equipment, and 19.7% stated that the sinks were dirty (Table 2).

**Table 2:** Nursing students’ opinions about hand hygiene (*n* = 240)

	Number	%
**The most effective method to prevent the transmission of infection in the hospital**		
Using gloves Hand hygiene Sterilization Isolation Asepsis-antisepsis Disinfection Other	14 103 69 8 40 3 3	5.8 42.9 28.7 3.3 16.7 1.3 1.3
**The best method for drying hands**		
Hand dryer Disposable paper towel Personal towel Common towel A clean sheet of paper	22 198 15 0 5	9.2 82.4 6.3 0 2.1
**Receiving training on hand hygiene**		
Yes No	185 55	77.1 22.9
**Need for hand hygiene training**		
Yes No	58 182	24.2 75.8
**Hand hygiene equipments in daily life**		
Only water Shared bar soap and water My own bar of soap and water Water and general liquid soap Water and my own liquid soap Alcohol-based hand antiseptic Alcohol	0 18 21 143 55 0 3	0 7.5 8.8 59.6 22.8 0 1.3
**Daily hand hygiene frequency**		
1–4 times a day 5–8 times a day 9–12 times a day 13–17 times a day 18–30 times a day	15 59 87 57 22	6.3 24.5 36.2 23.8 9.2
**Drying hands after washing**		
Yes, definitely Occasionally No	175 65 0	72.9 27.1 0
**Presence of dermatological problems on hands**		
Yes No	33 207	13.7 86.3
**Barriers to hand hygiene**		
No barriers Dirty sinks Lack of sinks/ faulty faucets Lack of hand hygiene equipment (soap, paper towels, etc.) Lack of time	76 47 22 87 6	31.9 19.7 9.2 36.6 2.6

### Mean scores of the SAHBTPB

The mean total score of the participating nursing students on the scale was 147.5 ± 14.0 (minimum-maximum: 94–176). In this study, the Cronbach’s alpha value of the scale was found to be 0.93. The sub-dimension mean scores were as follows: “beliefs about the consequences of hand hygiene” 45.5 ± 4.5 (min-max: 31–56), “subjective norm” 3.3 ± 0.6 (min-max: 1–4), “normative beliefs” 20.8 ± 3.0 (min-max:12–24), “perceived control” 5.9 ± 1.0 (min-max: 2–8), “attitude” 20.0 ± 2.6 (min-max: 13–24), “intention” 12.1 ± 2.5 (min-max: 4–16), and “knowledge” 23.4 ± 1.9 (min-max: 11–24) (Table 3).

**Table 3:** Nursing students’ mean scores on the scale and its sub-dimensions

Sub-dimensions	Number of items	X̅ ± SD	Distribution range	Lowest-Highest scores	Cronbach Alpha Coefficient
**Beliefs about the consequences of hand hygiene**	14	45.5 ± 4.5	14–56	31.00–56.00	0.75
**Subjective norm**	1	3.3 ± 0.6	1–4	1.00–4.00	n/a
**Normative beliefs**	6	20.8 ± 3.0	6–24	12.00–24.00	0.94
**Control beliefs**	5	16.2 ± 2.4	5–20	8.00–20.00	0.79
**Perceived control**	2	5.9 ± 1.0	2–8	2.00–8.00	0.79
**Attitude**	6	20.0 ± 2.6	6–30	13.00–24.00	0.71
**Intention**	4	12.1 ± 2.5	4–16	4.00–16.00	0.82
**Knowledge**	8	23.4 ± 1.9	8–24	11.00–24.00	0.85
**Total**	46	147 ± 14.0	46–176	94.00-176.00	0.93

X̅ ±SD = Total score mean ± Standard deviation, n/a = Not assessed

When examining the mean scores based on the items on the scale, the statement “If I regularly follow hand hygiene procedures at the hospital, my hands may dry out and my skin may become irritated’’ received the lowest mean score of 2.60 ± 0.75. The statement “If I follow the hand hygiene procedure regularly in the hospital, I feel relieved” had the highest mean score of 3.64 ± 0.53.

We conducted a correlation analysis to measure the degree of relationship between nursing students’ SAHBTPB and its sub-dimensions and their effects on hand hygiene intentions. We used the standard developed by Schober & Schwarte (2018) to evaluate the strength of the relations, in which coefficients in the range 0.00–0.19 represent a very weak correlation; those within 0.20–0.39, a weak correlation; those within 0.40–0.69, a moderate correlation; those within 0.70–0.89, a strong correlation; and those within 0.90–1.00, a very strong correlation [32]. Our results indicated a significant positive and high-level relationship between the total mean scores of the scale and the mean scores of the intention sub-dimension (*r* = .702, *p* < .001) and control beliefs sub-dimension (*r* = .620, *p* < .001). The intention sub-dimension demonstrated a significant positive and low-level relationship with the subjective norm (*r* = .298, *p* < .001). It also showed a significant positive and moderate-level relationship with perceived behavioral control (*r* = .487, *p* < .001), attitude (*r* = .485, *p* < .001), beliefs about the outcomes of hand hygiene (*r* = .447, *p* < .001), and normative beliefs (*r* = .401, *p* < .001) sub-dimension mean scores. However, no significant relationship was observed between intention and knowledge sub-dimension mean scores (*r* = .108, *p* > .05). A positive and significant correlation was identified between the students’ total scale mean scores and all sub-dimension mean scores (*p* < .001) (Table 4). The correlations between the knowledge sub-dimension and the subjective norm (*r* = .088, *p* > .05), control belief (*r* = .062, *p* > .05) and intention sub-dimensions (*r* = .108, *p* > .05) were positive but not statistically significant (Table 4).

**Table 4:** Correlation analysis of the total SAHBTPB scores and sub-dimensions (*N* = 240)

			Total scale score Total score	Beliefs about the consequences of hand hygiene	Subjective norm	Normative beliefs	Control beliefs	Perceived control	Attitude	Intention	Knowledge	Item	X̅ *±*SD	Cronbach Alpha Coefficient
**Sub-dimensions**	**Total scale score**	Correlation Coefficient	1.000									46	147.5 ± 14.0	0.93
		Sig. (2-tailed)	.											
	**Beliefs about the consequences of hand hygiene**	Correlation Coefficient	0.856^**^	1.000								14	45.5 ± 4.5	0.75
		Sig. (2-tailed)	*p* < .001	.										
	**Subjective norm**	Correlation Coefficient	0.646^**^	0.550^**^	1.000							1	3.3 ± 0.6	n/a
		Sig. (2-tailed)	*p* < .001	*p* < .001	.									
	**Normative beliefs**	Correlation Coefficient	0.773^**^	0.613^**^	0.662^**^	1.000						6	20.8 ± 3.0	0.94
		Sig. (2-tailed)	*p* < .001	*p* < .001	*p* < .001	.								
	**Control beliefs**	Correlation Coefficient	0.824^**^	0.625^**^	0.536^**^	0.612^**^	1.000					5	16.2 ± 2.4	0.79
		Sig. (2-tailed)	*p* < .001	*p* < .001	*p* < .001	*p* < .001	.							
	**Perceived control**	Correlation Coefficient	0.561^**^	0.400^**^	0.393^**^	0.325^**^	0.505^**^	1.000				2	5.9 ± 1.0	0.79
		Sig. (2-tailed)	*p* < .001	*p* < .001	*p* < .001	*p* < .001	*p* < .001	.						
	**Attitude**	Correlation Coefficient	0.791^**^	0.588^**^	0.459^**^	0.535^**^	0.597^**^	0.411^**^	1.000			6	20.0 ± 2.6	0.71
		Sig. (2-tailed)	*p* < .001	*p* < .001	*p* < .001	*p* < .001	*p* < .001	*p* < .001	.					
	**Intention**	Correlation Coefficient	0.702^**^	0.477^**^	0.298^**^	0.401^**^	0.620^**^	0.487^**^	0.485^**^	1.000		4	12.1 ± 2.5	0.82
		Sig. (2-tailed)	*p* < .001	*p* < .001	*p* < .001	*p* < .001	*p* < .001	*p* < .001	*p* < .001	.				
	**Knowledge**	Correlation Coefficient	0.281^**^	0.197^**^	0.088	0.179^**^	0.062	0.127^*^	0.255^**^	0.108	1.000	8	23.4 ± 1.9	0.85
		Sig. (2-tailed)	*p* < .001	0.002	0.173	0.005	0.336	0.049	*p* < .001	0.094	.			

X̅ ±SD = Total score mean ± Standard deviation n/a = Not assessed **Correlation is significant at the 0.01 level (2-tailed). * Correlation is significant at the 0.05 level (2-tailed)

According to students’ gender, hand hygiene scale overall total mean scores (*p* = .012), beliefs about the consequences of hand hygiene *(p* = .027), subjective norm (*p* = .037), attitude (*p* = .003) and knowledge (*p* < .001) a statistical difference was detected between the sub-dimension scores. No statistical difference was observed between normative beliefs, control beliefs, perceived control and intention sub-dimension mean scores according to gender. The total scale score and sub-dimension mean scores were higher in women than in men (Table 5).

**Table 5:** SAHBTPB total score and sub-dimension score mean analysis according to students’ demographic characteristics

Demographic Features	Beliefs about the consequences of hand hygiene	Subjective norm	Normative beliefs	Control beliefs	Perceived control	Attitude	Intention	Knowledge	Total score mean
**Gender** Man Women	44.4 *±* 4.5 45.9 *±* 4.5 z:-2.205, *p* < .05	3.1 *±* 0.6 3.3 *±* 0.6 z:-2.091, *p* < .05	20.3 *±* 3.2 21.1 *±* 2.9 z:-1.153, *p* > .05	15.9 *±* 2.3 16.3 *±* 2.5 z:-1.153, *p* > .05	5.9 *±* 0.9 6.0 *±* 1.1 z:-0.890, *p* > .05	19.1 *±* 2.8 20.3 *±* 2.5 z:-2.976, *p* < .05	12.0 *±* 2.4 12.2 *±* 2.5 z:-0.306, *p* > .05	22.6 *±* 3,0 23.7 *±* 0.9 z:-3.677, *p* < .001	143.7 *±* 13.0 149.2 *±* 13.8 z:-2.520, *p* < .05
**Graduated school** Health college High school Associate degree	47.6 *±* 2.6 45.4 *±* 4.5 45.4 *±* 4.7 KW: 1.004, *p* > .05	3.4 *±* 0.8 3.3 *±* 0.6 3.5 *±* 0.5 KW: 1.485, *p* > .05	22.0 *±* 2.8 20.8 *±* 3.0 21.6 *±* 2.8 KW: 1.784, *p* > .05	16.4 *±* 1.1 16.2 *±* 2.5 16.3 *±* 2.1 KW: 0.078, *p* > .05	6.4 *±* 0.8 5.9 *±* 1.0 6.3 *±* 0.7 KW: 1.875, *p* > .05	21.0 *±* 2.7 20.0 *±* 2.6 19.7 *±* 3.1 KW: 0.613, *p* > .05	11.2 *±* 1.3 12.1 *±* 2.5 12.4 *±* 1.8 KW: 0.866, *p* > .05	23.8 *±* 0.4 23.4 *±* 1.9 23.6 *±* 0.7 KW: 0.425, *p* > .05	151.8 *±* 8.5 147.4 *±* 14.2 149.3 *±* 12.1 KW: 0.564, *p* > .05
**Level of grade** First grade Second grade Third grade Fourth grade	43.9 *±* 4.5 45.8 *±* 4.3 46.0 *±* 4.1 47.5 *±* 4.2 KW:21.378, *p* < .001	3.1 *±* 0.7 3.3 *±* 0.5 3.4 *±* 0.5 3.4 *±* 0.5 KW:12.056, *p* < .05	20.0 *±* 3.0 21.4 *±* 2.9 21.3 *±* 2.7 21.4 *±* 3.0 KW:9.740, *p* < .05	15.7 *±* 2.4 16.2 *±* 2.1 15.5 *±* 2.3 17.7 *±* 2.4 KW:21.922, *p* < .001	5.6 *±* 1.0 6.0 *±* 0.9 5.9 *±* 0.8 6.6 *±* 0.9 KW:28.885, *p* < .001	19.2 *±* 2.6 20.0 *±* 2.6 20.8 *±* 2.2 20.6 *±* 2.8 KW:12.494, *p* < .05	12.0 *±* 2.3 12.2 *±* 2.5 10.7 *±* 2.2 13.7 *±* 2.5 KW:28.760, *p* < .001	22.8 *±* 2.6 23.9 *±* 0.2 23.8 *±* 0.4 23.3 *±* 1.9 KW:18.887, *p* < .001	142.5 *±* 13.7 149.2 *±* 13.1 147.8 *±* 12.6 154.6 *±* 13.6 KW:21.857, *p* < .001
**Receiving training on hand hygiene** Yes No	45.9 *±* 4.5 44.0 *±* 4.2 z: -2.868, *p* < .05	3.3 *±* 0.5 3.0 *±* 0.7 z: -2.592, *p* < .05	21.0 *±* 2.9 20.3 *±* 3.2 z: -1.185, *p* > .05	16.3 *±* 2.4 15.6 *±* 2.5 z: -1.632, *p* > .05	6.1 *±* 1.0 5.4 *±* 1.0 z: -4.095, *p* < .001	20.2 *±* 2.6 19.2 *±* 2.6 z: -2.381, *p* < .05	12.2 *±* 2.6 12.0 *±* 2.2 z: − 0.412, *p* > .05	23.6 *±* 1.6 22.8 *±* 2.4 z:-3.229, *p* < .05	148.9 *±* 14.1 142.8 *±* 12.5 z: -2.891, *p* < .05
**Dermatological problems** Yes No	46.3 *±* 3.6 45.3 *±* 4.6 z: − 0.985, *p* > .05	3.3 *±* 0.5 3.3 *±* 0.6 z: − 0.541, *p* > .05	21.7 *±* 2.6 20.7 *±* 3.0 z: -1.637, *p* > .05	16.4 *±* 2.1 16.1 *±* 2.5 z: − 0.450, *p* > .05	5.9 *±* 0.9 6.0 *±* 1.0 z: − 0.075, *p* > .05	20.1 *±* 2.4 19.9 *±* 2.7 z: − 0.117, *p* > .05	12.7 *±* 2.2 12.0 *±* 2.5 z: − 0.1.288, *p* > .05	23.5 *±* 1.5 23.4 *±* 1.9 z:-0.350, *p* > .05	150.2 *±* 11.6 147.1 *±* 14.3 z: -1.082, *p* > .05

Z = Mann-Whitney Test, KW = Kruskal Wallis Test

There was a statistical difference was found in the hand hygiene total scale mean scores among nursing students based on their grades (*p* < .001). Furthermore, concerning grades, significant differences were determined in beliefs about the consequences of hand hygiene (*p* < .001), subjective norm (*p* = .007), normative beliefs (*p* = .021), control beliefs (*p* < .001), perceived control (*p* < .001). Significant differences were also identified in attitude (*p* = .006), intention (*p* < .001), and knowledge (*p* < .001) sub-dimension mean scores. Generally, fourth-grade students had higher scale mean scores compared to other grades. However, a statistically significant difference was defined only between the scale mean scores of fourth and first-grade students (*p* < .001) (not shown in the data table).

Nursing students who received hand hygiene education showed statistically significant differences in hand hygiene total scale score means (*p* = .004), as well as beliefs about the consequences of hand hygiene (*p* = .004), subjective norm (*p* = .010), perceived control (*p* < .001), attitude (*p* = .017), and knowledge (*p* = .001) sub-dimension scores, compared to those who didn’t receive training. The scores were higher in students who received training than in those who did not (Table 5).

A significant yet weak correlation was found between the hand hygiene total scale mean scores and sub-dimension mean scores according to the students’ daily hand hygiene frequency *(p* < .001). Moreover, there was no statistically significant difference in the total scale scores and sub-dimension scores between students reporting dermatological problems on their hands and those who did not (*p* > .05).

## Discussion

Approximately 70% of the students participating in the study were female, and the mean age was around 21. Similarly, in a study by Jeong and Kim (2016), the mean age of the nursing students was around 22, and 89% of them were female [26]. In a study by Zimmerman et al., (2020), almost all (93.7%) of the 225 nursing students were female, and 70% were in the 18–25 age range [22, 26]. These studies observed that the sample groups mostly consist of female students, and their mean ages were similar.

### Views and practices regarding hand hygiene

Nearly half of the students (42.9%) reported hand hygiene as the most effective method in preventing HAIs. A study examining the knowledge and attitudes of nursing students towards HAIs reported that almost all participants indicated hand hygiene as the most effective method in preventing HAIs [33]. Similar findings were reported in the study by Zimmerman et al., (2020) [22]. Our study’s findings are consistent with those of other studies, highlighting the widespread acknowledgment among nursing students that hand hygiene is paramount in preventing HAIs.

While three-quarters of the students stated that they received hand hygiene training, 24.2% expressed the need for retraining despite having received training. In other studies, almost all nursing students were reported to have received hand hygiene training [26, 34–36]. However, it is noteworthy that in this study, some students did not mention receiving training on hand hygiene despite it being included in the curriculum. This situation may be related to the low awareness of students about hand hygiene, individual differences, and forgetting that they have received training on the subject.

In clinical practice, students identified barriers to hand hygiene such as lack of materials, dirty, faulty, or insufficient number of sinks. In the literature, barriers to hand hygiene include lack of paper towels or soap, glove usage, heavy workload, lack or absence of role models, time constraints, and skin problems [35, 37, 38]. The barriers to hand hygiene identified in our study generally resemble those mentioned in the literature. Therefore, common action plans should be developed and implemented to address these barriers.

### Data obtained from the SAHBTPB

Despite a low reported rate of students receiving hand hygiene training prior to the implementation of the scale, it was found that students scored significantly high on the hand hygiene behavior scale. This situation may be related to the compulsory teaching of hand hygiene at both theoretical and practical levels in each class. Additionally, the increase in the time spent in clinical settings as the grade level increases, along with increased awareness due to the recent Covid-19 pandemic, may have contributed to this outcome. In the correlation analysis conducted, a positive relationship was found between the students’ total SAHBTPB scores and the scores of all sub-dimensions.

Sub-dimensions other than the knowledge sub-dimension, which were thought to influence intention motivationally, were correlated with the intention sub-dimension. Sub-dimensions most strongly correlated with intention were perceived behavioral control, control beliefs, and attitude. Among the sub-dimensions, it was determined that control beliefs were the sub-dimension most strongly associated with intention. Consistent with our study, O’Boyle et al., (2001) found a positive and significant relationship between the intention sub-dimension and control beliefs. This indicates that students are proficient in controlling factors that hinder hand hygiene. In the study reported also that the theory’s variables predicted intention but were insufficient to predict actual behavior [9]. In a study conducted in the United States, a significant relationship was found between nurses’ subjective norms and perceived behavioral control sub-dimensions and their self-reported and observed hand hygiene behaviors [27]. A study in South Korea reported that when nursing students’ perceived control belief sub-dimension scores were high, observed hand hygiene compliance was also higher [26]. A study conducted in Thailand reported a significant positive relationship between attitude, perceived behavioral control, TPB scale total scores, and observed hand hygiene compliance among nurses and nursing students [39]. O’Boyle et al., (2001), Sin & Rochelle (2022), and White et al., (2015) stated that in studies conducted with nurses, TPB could predict nurses’ self-reported hand hygiene behaviors [9, 28, 40]. In our study, only TPB sub-dimensions were examined, and it is seen that there is a significant relationship between the overall scale total scores and the total scores of all sub-dimensions in our findings and other studies. In this context, it is possible to say that the scale is successful in predicting the determinants of hand hygiene behavior in advance. Factors influencing hand hygiene behaviors include gender, role models, habits acquired during childhood, and handwashing facilities [30]. Sax et al., (2007) stated that demographic characteristics and past experiences contribute to the shaping of beliefs and behaviors related to hand hygiene [41]. In Kim & Oh’s (2015) study, students reported that negative role models observed during clinical practice and gaps in class and field negatively affected hand hygiene behaviors. Students expressed that the practices they experienced in clinical settings were quite different from what they learned in class [29].

In a study conducted with nursing students in Spain, it was reported that the use of alcohol-based hand sanitizers and knowledge of hand hygiene were significantly higher in female students, and there was a meaningful adherence to hand hygiene [42]. Karadağ et al., (2016) also indicated that the mean score they got from the hand hygiene scale was high in students. In study, the mean scale scores of male nurses were higher than those of female nurses, whereas the mean scale scores of female students were higher than those of male students. In contrast, our study revealed a higher mean score among female students [43]. Similar results have been reported in some other studies, suggesting that male students may have weaker performance in hand hygiene compared to their female counterparts [30, 44, 45]. Our study results align with previous findings, and this similarity could be associated with gender roles, the higher awareness of female students regarding the importance of hand hygiene, and the larger number of female students.

Hand hygiene compliance can be influenced by factors such as hand hygiene education, participation in campaigns, and existing knowledge [17, 40, 46]. However, in our study, there was no significant relationship between hand hygiene intention and the knowledge sub-dimension scores. Similar findings were reported by Jeong & Kim (2013) and Yoon & Kim (2013), stating that hand hygiene knowledge does not necessarily impact actual hand hygiene behavior [47, 48]. Jeong & Kim (2013) associated this with the indifference or poor role modeling of administrators and clinical nurses during clinical practices [47]. In a study by O’Boyle et al., (2001), TPB, including intention, was found to be significantly related to self-reported hand hygiene compliance but not congruent with observed hand hygiene performance [9, 49]. Despite having well-structured hand hygiene knowledge, practical variables in our students might be more determinant of hand hygiene intention than theory.

In our study, there were statistically significant differences in hand hygiene scale scores among students based on their academic years. Particularly, fourth-grade students had significantly higher scores compared to first-grade students. In a study by Öncü et al., (2019), first-grade students were found to use hand sanitizers more than fourth-grade students [35], and Amin et al., (2013) discovered that medical students, despite having high knowledge levels, did not adhere to hand hygiene rules [50]. In our study, the increase in theoretical and practical courses with higher academic years might have contributed to this difference. Additionally, the longer clinical experiences and providing care in high-risk infection areas during internship for fourth-grade students could have increased their hand hygiene knowledge and awareness. In conclusion, it is considered that practice-related variables play a more dominant role than knowledge in increasing hand hygiene compliance. Therefore, it is crucial for mentor teachers, clinicians, and nurse managers to collaborate in eliminating practice-related negative factors.

### Limitations and future direction

Our study has some limitations. Firstly, the information regarding the hand hygiene practices of student nurses relies on their self-reports, restricting the results to the responses of this particular student group. Secondly, the lack of observation of students’ hand hygiene compliance in clinical settings and the limitation to nursing students from only one university are other constraints. The third limitation of our study may arise from the use of an even-numbered Likert scale in the SAHBTPB instrument, which may introduce a bias by not providing a neutral option. However, the validation and adequacy of its psychometric properties support its reliability and validity despite this limitation. The inability to assess the impact of geographical and educational environment differences on the hand hygiene behavior of nursing students is the fourth limitation. Therefore, it is recommended that future research incorporates studies with broader and more diverse sample groups, combining self-report and observational methods.

### Conclusion and recommendations

Despite the awareness of the importance of hand hygiene, there are still unknown psychosocial factors that motivate healthcare professionals to perform hand hygiene behavior. In this study using the TPB, the intentions of nursing students towards hand hygiene have been acknowledged as a determining factor in behavior. Therefore, efforts have been made to identify factors influencing the intention that determines hand hygiene behavior. Analysis of the data obtained from the students’ responses revealed that gender, grade level, and receiving training on hand hygiene had a positive and direct impact on the total score obtained from the scale. Before nursing candidates start their professional careers, applying behavioral theories to instill hand hygiene behavior is recommended. Recognizing that factors such as knowledge, beliefs, and attitudes influence hand hygiene behavior, identifying problems related to these factors, and planning and organizing educational interventions targeting these problems during training are believed to significantly improve learning outcomes. Additionally, supportive hand hygiene areas should be established in clinical training settings to ensure correct application of hand hygiene techniques and enhance compliance.

## Data Availability

The datasets generated and/or analysed during the current study are not publicly available due to privacy and confidentiality concerns but are available from the corresponding author on reasonable request.
